# Cholesterol Hydroperoxide Co-trafficking in Testosterone-generating Leydig Cells: GPx4 Inhibition of Cytotoxic and Anti-steroidogenic Effects

**DOI:** 10.1007/s12013-023-01194-5

**Published:** 2023-11-23

**Authors:** Pawel Pabisz, Jerzy Bazak, Michal Sabat, Albert W. Girotti, Witold Korytowski

**Affiliations:** 1 Department of Biophysics, Jagiellonian University, Krakow, Poland; 2 Department of Biochemistry, Medical College of Wisconsin, Milwaukee, WI 53226 USA

**Keywords:** Steroidogenesis, Testosterone, Cholesterol Hydroperoxide, Mitochondria, Lipid Peroxidation, GPx4

## Abstract

Trafficking of intracellular cholesterol (Ch) to and into mitochondria of steroidogenic cells is required for steroid hormone biosynthesis. This trafficking is typically mediated by one or more proteins of the steroidogenic acute regulatory (StAR) family. Our previous studies revealed that 7-OOH, a redox-active cholesterol hydroperoxide, could be co-trafficked with Ch to/into mitochondria of MA-10 Leydig cells, thereby inducing membrane lipid peroxidation (LPO) which impaired progesterone biosynthesis. These negative effects of 7-OOH were inhibited by endogenous selenoperoxidase GPx4, indicating that this enzyme could protect against 7-OOH-induced oxidative damage/dysfunction. In the present study, we advanced our Leydig focus to cultured murine TM3 cells and then to primary cells from rat testis, both of which produce testosterone. Using a fluorescent probe, we found that extensive free radical-mediated LPO occurred in mitochondria of stimulated primary Leydig cells during treatment with liposomal Ch+7-OOH, resulting in a significant decline in testosterone output relative to that with Ch alone. Strong enhancement of LPO and testosterone shortfall by RSL3 (a GPx4 inhibitor) and reversal thereof by Ebselen (a GPx4 mimetic), suggested that endogenous GPx4 was playing a key antioxidant role. 7-OOH in increasing doses was also cytotoxic to these cells, RSL3 exacerbating this in Ebselen-reversable fashion. Moreover, GPx4 knockdown increased cell sensitivity to LPO with reduced testosterone output. These findings, particularly with primary Leydigs (which best represent cells in intact testis) suggest that GPx4 plays a key protective role against peroxidative damage/dysfunction induced by 7-OOH co-trafficking with Ch.

## Introduction

Free (unesterified) cholesterol (Ch) is required for the initiation of steroid hormone synthesis in steroidogenic organs, including the ovary, testis, placenta, and adrenal gland [1–3]. There are several possible sources of this Ch, including (i) import of Ch- and cholesteryl ester-bearing low density lipoprotein (LDL) via its cell surface receptor, (ii) hydrolysis of cholesteryl esters in endogenous lipid droplets, (iii) removal from the Ch-rich plasma membrane, and (iv) de novo synthesis in the endoplasmic reticulum [3–5]. Initiation of hormone synthesis occurs in mitochondria (Mito) via hydroxylation and cleavage of the Ch side chain to give pregnenolone. This reaction is catalyzed by cytochrome P450 side-chain cleavage enzyme (P450scc/Cyp11A1) located on the Mito inner membrane [2, 6]. Trafficking proteins of the steroidogenic acute regulatory (StAR) family play a major role in steroid hormone synthesis by transporting Ch to and into Mito [5, 7, 8]. Each of these proteins contains a C-terminal START domain that binds a single Ch molecule in highly specific fashion [9, 10]. StarD1, the family prototype on the inner membrane, acts in conjunction with several other proteins to effect translocation of incoming Ch to the inner membrane for processing by P450scc [7, 8, 11]. StarD1 has at least three structural homologs (StarD4-D6), which lack organelle-targeting sequences [7, 12], relegating their functionality to the cytosol. It has been proposed that StarD4, for example, moves Ch through the cytosol to the outer Mito membrane, where resident StarD1 and associated proteins then move it to the inner membrane for conversion to pregnenolone [7, 12–14]. The latter then enters the smooth endoplasmic reticulum and is metabolized to progesterone and thence to testosterone and other androgens in the testis, estrogens in the ovary, or glucocorticoids and mineralocorticoids in the adrenal cortex [1, 2].

There is growing evidence that oxidized LDL (oxLDL) generated by persistent oxidative stress can impair male fertility by decreasing testosterone output of the testis [15–17]. Ch and other unsaturated lipids are susceptible to oxidative modification under such conditions, the overall process being known as lipid peroxidation (LPO). Sterol ring hydroperoxides of Ch (ChOOHs), like fatty acyl hydroperoxides of phospholipids and glycolipids, are early intermediates of LPO [18, 19]. The epimeric ChOOHs 3β-hydroxycholest-5-ene-7α-hydroperoxide (7α-OOH) and 3β-hydroxycholest-5-ene-7β-hydroperoxide (7β-OOH), are key species generated by free radical-mediated Ch oxidation [20]. Like all lipid hydroperoxides (LOOHs), 7α/7β-OOH (collectively referred to as 7-OOH) are susceptible to iron-catalyzed one-electron reduction to oxyl radicals (7-OO^•^) which can induce chain LPO that exacerbates lipoprotein or membrane peroxidative damage [19, 21]. Alternatively, 7-OOH may undergo enzyme-catalyzed two-electron reduction to relatively harmless alcohols (7-OH), resulting in restriction of peroxidative damage [19]. Selenium-containing glutathione peroxidase-type 4 (GPx4) can catalyze the reductive inactivation of phospholipid hydroperoxides (PLOOHs) as well as ChOOHs, glutathione (GSH) acting as a co-substrate [22, 23]. GPx4 appears to be the only known enzyme capable of detoxifying ChOOHs in membrane or lipoprotein environments [22]. This distinguishes GPx4 from its more abundant mammalian homolog, GPx1, which acts on H_2_O_2_ and fatty acid hydroperoxides, but not on PLOOHs or ChOOHs [24].

Early studies by others [25–27] revealed that low levels of 7-OOH are present in oxLDL isolated from human serum. 7-OOH levels were significantly higher in oxLDL obtained from diabetic subjects or from atherosclerotic lesions [26, 27], and there was a strong indication that ongoing oxidative stress was responsible. It was also found that the cytotoxic potency of isolated oxLDL correlated directly with its content of 7-OOH [26, 27]. The 7-peroxide was also detected in Cu(II)-treated human LDL after modest photooxidation priming [28]. Knowing this, we hypothesized earlier [29] that 7-OOH from oxLDL can be co-trafficked with Ch to/into Mito of steroidogenic cells, thereby triggering membrane damage/dysfunction due to one-electron turnover and initiation of chain LPO. Strong initial support for this hypothesis was obtained by showing that Mito of dibutyryl cAMP-stimulated testicular MA-10 Leydig cells underwent a rapid loss of trans-membrane potential during exposure to 7-OOH-bearing liposomes; concurrently, progesterone production by these cells was markedly reduced [29]. StarD1 knockdown prior to cell stimulation resulted in far less (i) peroxide uptake (assessed with [^14^C]7-OOH), (ii) loss of membrane potential, and (iii) diminished progesterone yield. All these effects were consistent with StarD1’s ability to deliver 7-OOH as well as Ch [29]. Prolonged exposure to 7-OOH at elevated concentrations also killed cells via intrinsic apoptosis. Unlike 7-OOH, *tert*-butyl hydroperoxide (*t*-BuOOH) at comparable levels did not damage MA-10 Mito to any significant extent or reduce progesterone yield [29]. Collectively, these findings supported the idea that under oxidative stress, steroid hormone synthesis can be impaired by Mito damage due to ChOOH/7-OOH delivery via a natural Ch trafficking pathway. More recent studies revealed that stimulated MA-10 cells internalized more 7-OOH via StarD1 than non-stimulated controls and exhibited greater mitochondrial membrane LPO due to one-electron turnover of 7-OOH [30]. Reduced progesterone yield following membrane damage/dysfunction was exacerbated when a GPx4 inhibitor was present, suggesting that GPx4 was functioning protectively by inactivating 7-OOH and/or downstream LOOHs [30]. This was the first known evidence for GPx4 acting as an antioxidant to combat oxidative stress impairment of steroidogenesis.

In the present study, we advanced from tumor-derived MA-10 cells, which are limited to progesterone synthesis, to TM3 and then primary Leydig cells, which metabolize Ch completely to the androgen testosterone. Our findings with primary cells, which are most closely representative of the intact testis, confirm that GPx4 plays a key role in protecting this organ against damage/dysfunction due to ChOOH co-trafficking under pathophysiologic pro-oxidant conditions. It is important to point out that the mammalian testis expresses relatively high levels of GPx4, and a strong positive correlation between active GPx4 status and human male fertility has been reported [31]. Given that LDL supplies most of Ch for testosterone synthesis in vivo [2, 32, 33], we used 7-OOH-containing liposomes to simulate natural oxLDL which carries this peroxide [25, 26].

## Materials and Methods

### General Materials

Leydig TM3 cells were obtained from ATCC (UK). Merck supplied the dibutyryl cyclic AMP (Bt2cAMP), Neutral Red, GPX4 inhibitor RSL3, Ebselen, horse serum, FBS, BSA, Hepes, Penicillin/Streptomycin, Luteinizing Hormone (LH), human Chorionic Gonadotropin (hCG), Percoll, Collagenase D, Dispase, DNase I, X-TremeGENE transfection reagent, Insulin-Transferrin-Sodium Selenite Supplement (TIS), Complete Mini (a Roche mixture of protease inhibitors) and Dulbecco’s modified Eagle’s/Ham’s nutrient F12 (DME/F12) medium. 1-Palmitoyl-2-oleoyl-sn-glycero-3-phosphocholine (POPC) and cholesterol were obtained from Avanti Polar Lipids. ThermoFisher Scientific provided M-199 medium, Opti-MEM medium, Soybean Trypsin Inhibitor (STI), DPBS, HBSS, EGF, C11-BODIPY(581/591), Mitotracker Deep Red, and primary rabbit antibodies against StarD1 and StarD4. A testosterone assay kit and primary rabbit antibodies against GPx4 and β-actin were obtained from Abcam. A horseradish peroxidase-conjugated IgG secondary antibody was from Cell Signaling Technology. Gel electrophoresis consumables were from BioRad. 7-OOH was prepared by dye-sensitized photoperoxidation of Ch, as described [34]. Stock solutions of 7-OOH in 2-propanol were standardized for hydroperoxide content by iodometric analysis [22] and stored at −20 °C.

### Animals and Isolation of Primary Leydig Cells

Adult male Sprague-Dawley rats, 90–120 days old and weighing 350–450 g, were sacrificed by decapitation. Testes were removed immediately and placed on ice in pH 7.2 M-199 medium containing STI (25 μg/ml), Penicillin (100 U/ml), and Streptomycin (0.1 mg/ml). Primary Leydig cell isolation was carried out as described [35, 36] with some modifications. To increase the yield and purity of isolated cells, each testis was cleared of fat, and the vasculature was perfused (under a dissecting microscope) with 0.8 ml of M-199 containing STI (25 μg/ml), Collagenase D (1 mg/ml), and Dispase (0.08 mg/ml), using a 0.3 × 13 mm needle. After perfusion testes were decapsulated and collected in fresh, ice-cold M-199 without collagenase. Batches of 8–10 decapsulated testes were placed in a T75 tissue culture flask containing 20 ml fresh M-199 and incubated at 34 °C for 10 min. At the end of this incubation, 20 ml of M-199 containing STI (25 μg/ml), Collagenase D (0.5 mg/ml), Dispase (0.04 mg/ml) and DNAse I (0.05 mg/ml) was added, and incubation was continued horizontally in a shaking water bath at 34 °C for ~1 h. When dissociated parenchyma appeared loose, the incubation was terminated and 80 ml collagenase-free M-199 was added to each flask. After the seminiferous tubule mass sedimented, supernatant was filtered through 70 μm nylon mesh and the dissociated cells were pelleted by centrifugation for 10 min at 250 × *g*. The recovered pellet was resuspended in 2 ml of M-199 containing STI (25 μg/ml), and carefully layered on the top of a discontinuous Percoll gradient prepared in 50 ml Falcon tubes by layering Percoll solutions (10 ml each) with densities 1.090, 1.063, 1.054, 1.040 g/ml. The tubes were centrifuged at 800 × *g* for 40 min at room temp. The fraction between 1.090 and 1.063 g/ml (approx. 6 ml) was collected, diluted to 50 ml with M-199 containing STI (25 μg/ml), Penicillin (100 U/ml), and Streptomycin (0.1 mg/ml), and centrifuged at 350 × *g* for 15 min. The pellet was resuspended and washed twice in DMEM-F12 containing 0.1% BSA to remove the remaining Percoll [37, 38]. Typical yield or primary Leydig cells obtained by the described procedure was approximately 1 × 10^6^ cells per testis. The purity checked by microscopic examination of the final cell fraction (Leydig cells being polyhedral in shape with a prominent nucleus and distinct nucleus) was in the range of 85–89%.

### Cell Culture

Leydig cells were plated at a density of ~3–6 × 10^4^/cm^2^ in DMEM-F12 + TIS + EGF + 0.1% BSA and cultured at 34 °C, under humidified atmosphere of 7% CO_2_ in air (conditions similar as those described by Browning et al. [39]. Seeding densities were as follow: 6-well plates/35 mm dishes 3–5 × 10^5^/well (for WB), 12-well plates 1.2 × 10^5^/well (testosterone production), 24-well plates 0.6–1 × 10^5^/well (testosterone production, lipids peroxidation), and 48-well plates 0.6 × 10^5^/well (cytotoxicity).

### GPX4 Knockdown

Leydig cells were transfected with 50 nM small interfering RNA (siRNA) directed against GPX4 (Ambion, s131172), or a control nontargeting siRNA (MISSION siRNA Universal Negative Control #1) using the X-tremeGENE siRNA Transfection Reagent (Roche) according to manufacturer’s protocol. After a 20 h incubation period, the medium was replaced with LC medium for the next 48 h.

### Western Blot Analyses

Cells plated on 35 mm dishes, were stimulated/or not with 5 ng/ml LH in culture medium for 3 h. Subsequently, medium was replaced with fresh medium with 5 ng/ml LH but w/o TIS/BSA and SUV’s were introduced. After 24 h incubation, cells were rinsed with cold DPBS, lysed in RIPA lysing buffer with protease inhibitors, and pelleted by centrifugation. After analysis for total protein, samples were prepared in Laemmli sample buffer, loaded onto a 4–20% gradient gel, and analyzed by standard SDS-PAGE. Separated proteins were transferred to a membrane, blocked, and incubated overnight at 4 °C with StarD1 and GPx4 antibody at a supplier-recommended dilution. After washing, the blots were treated with a peroxidase-conjugated secondary antibody and analyzed, using Super-Signal West Pico chemiluminescence detection. Blots were also probed for β-actin as a loading control. Other details were as described previously [40].

### Assessment of Extent and Localization of Lipid Peroxidation by Fluorescence Microscopy

Cells in 24-wells plates with cover glass bottom were incubated with 5 ng/ml LH in the presence or absence of RSL-3 and/or Ebselen. After 6 h of incubation, MitoTracker Deep Red and C11-Bodipy581/591 were added at a final concentration 200 nM and 10 μM, respectively. Cells were labeled for 30–45 min, washed, and POPC/Ch/ChOOH (5:4:1 by mol) SUVs were introduced at 20 μM ChOOH concentration in bulk. After 2 h incubation, cells were investigated under a fluorescence microscope. Fluorescence signals were acquired using LED sources 625 nm and 470 nm for excitation, and bandpass filters for emission: 676/29 (MitoTracker), 605/15 (C11-Bodipy581/591) and 525/30 (oxidized C11-Bodipy581/591).

### Testosterone Determination

A competitive immune-enzymatic assay for determination of testosterone was obtained from Abcam and used as recommended by the supplier.

### Liposome Preparation

Small unilamellar liposomes (SUVs) consisting of POPC/Ch/7-OOH (1.0:0.8:0.2 by mol) or POPC/Ch (1.0:0.8 by mol) were prepared by an extrusion method [41], stored under argon at 4 °C, and used for experiments within one week after preparation.

### Cell Viability Assay

An in vitro toxicology assay kit involving Neutral Red for determining cell viability was used according to supplier recommendations.

### Statistical Analysis

The two-tailed Student’s *t*-test was used for determining the significance of perceived differences between experimental values; a *p* value > 0.05 was considered statistically insignificant.

## Results

### Sensitivity of TM3 Cells to 7-OOH Toxicity

**Fig. 1:**
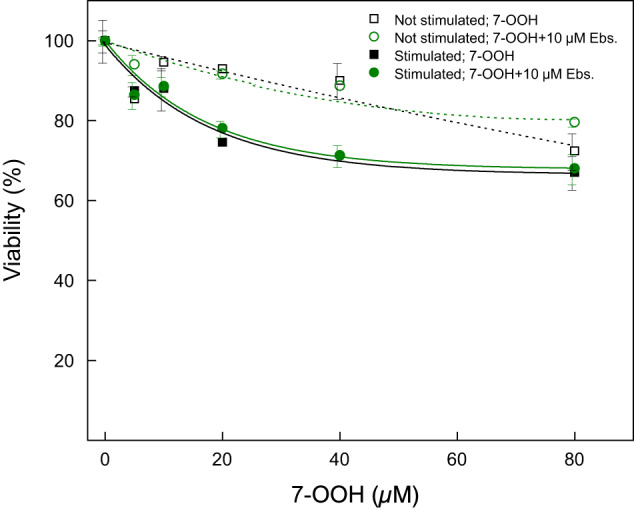
Effect of a GPx4 mimetic (Ebselen) on sensitivity of stimulated vs non-stimulated TM3 Leydig cells to a 7-OOH challenge. Cells at 60–70% confluency in 48-well plates were either not stimulated or stimulated with hCG (0.2 IU/ml) in serum-free DMEM-F12 medium. Ebselen (10 µM) was included in some wells, as indicated. After 12 h of incubation, POPC/Ch (5:4 by mol) or POPC/Ch/7-OOH (5:4:1 by mol) SUVs were added, the former serving as a control and latter giving the indicated concentrations of 7-OOH in bulk phase. After incubating for 24 h in the presence of 1% serum, cell viability was determined by Neutral Red assay. Plots show viability of (a) non-stimulated cells in the absence (·) vs. presence (o) of Ebselen, and (b) stimulated cells in the absence (■) vs. presence (●) of Ebselen – all as a function of 7-OOH concentration in bulk medium. Plotted values (means ± SEM, *n* = 3) indicate viability relative to a non-7-OOH-treated control

In an early experiment, we compared hCG-stimulated TM3 cells with non-stimulated counterparts for their sensitivity to 7-OOH-induced killing. As shown in Fig. 1, cell viability decreased with increasing concentration of SUV-Ch/7-OOH, but the drop-off was significantly more rapid for the stimulated cells, e.g. ~25% vs. ~5% a 20 µM 7-OOH. Previous studies with another Leydig cell type, MA-10, revealed that sterol trafficking protein StarD1 was strongly upregulated after cell stimulation [30], suggesting that this might account for the greater TM3 sensitivity after stimulation. Higher StarD1 levels would allow more 7-OOH to be delivered to TM3 mitochondria, thus inducing cyto-lethal damage as observed with MA-10 cells [30]. Ebselen, a synthetic organo-selenium compound with antioxidant/cytoprotective activity, is often used as a selenoperoxidase mimetic [42]. When added to either stimulated or non-stimulated cells, Ebselen provided no significant additional resistance to 7-OOH cytotoxicity (Fig. 1). This suggests that any endogenous protection against 7-OOH was maximal under the conditions used and could not be enhanced by Ebselen.

### Role of GPx4 in Protecting TM3 Cells Against 7-OOH Toxicity

**Fig. 2:**
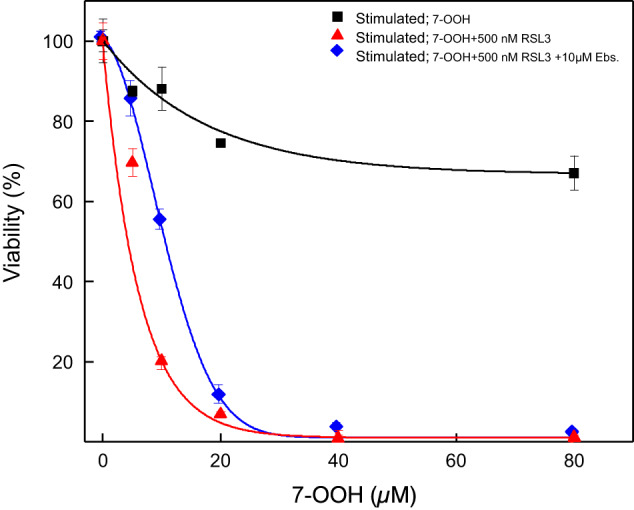
TM3 cell killing during exposure to liposomal 7-OOH: enhancement by RSL3 and partial reversal by Ebselen. Cells were stimulated with hCG as described in Fig. 1; some cell wells contained RSL3 (500 nM) alone or RSL3 plus Ebselen (10 µM). After incubating for 12 h, POPC/Ch/7-OOH (5:4:1 by mol) SUVs were added to give the indicated concentrations of 7-OOH in bulk phase. Controls were treated with serum plus POPC/Ch (5:4 by mol) SUVs. Neutral-Red-assessed cell viability was determined after 24 h of incubation. Plotted values are means ± SEM, *n* = 3

We asked whether endogenous GPx4 might provide TM3 cells with resistance against 7-OOH, given that this enzyme is known to detoxify ChOOHs [26, 27]. To examine this, we used RAS-selective lethal compound-3 (RSL3) as a GPx4 activity inhibitor [43, 44]. As shown in Fig. 2, RSL3 caused a dramatic increase in 7-OOH-induced killing of stimulated TM3 cells, e.g. ~20% viability vs. 70% viability at 10 µM 7-OOH. Knowing this, we asked whether Ebselen might be able to “rescue” these cells from the greater kill due to GPx4 inhibition. As seen in Fig. 2, Ebselen significantly reduced the extent of RSL3-enhanced cell killing - most likely by compensating, at least in part, for the GPx4 inactivation. Collectively, these results support the idea GPx4 plays a strong anti-ChOOH protective role in these Leydig cells.

### Effects of 7-OOH on testosterone output of TM3 cells

**Fig. 3:**
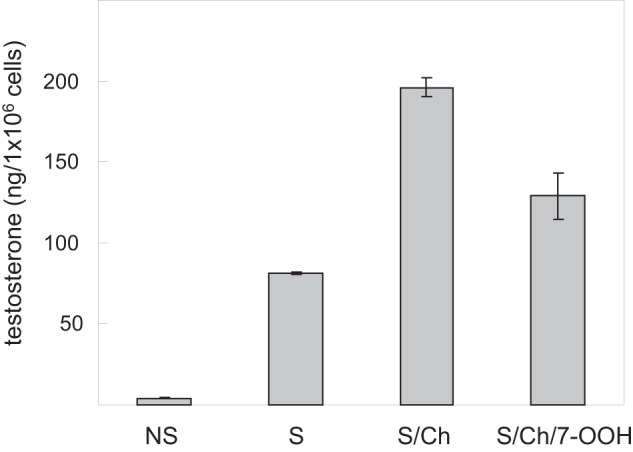
Effects of 7-OOH on testosterone production by stimulated TM3 cells. Cells that were not stimulated (NS) or hCG-stimulated (S) as described in Fig. 1 were exposed to POPC/Ch (5:4 by mol) SUVs (S/Ch) or POPC/Ch/7-OOH (5:4:1 by mol) SUVs (S/7-OOH). After 3 h of incubation, 150 µl of medium from each sample was analyzed for testosterone level using an ELISA assay. Plotted values are means ± SEM, *n* = 3

Unlike MA-10 cells, TM3 Leydig cells are enzymatically equipped to synthesize testosterone [45]. Therefore, we examined the effect of 7-OOH on testosterone generation, but reduced time of cell exposure to the peroxide to minimize its cytotoxic effects. As shown in Fig. 3, testosterone output of non-stimulated TM3 cells was negligible compared with stimulated counterparts (<5 ng/ml vs. 40 ng/ml). After incubation with Ch-containing SUVs, testosterone increased 25-fold to nearly 100 ng/ml. However, it fell dramatically to ~50 ng/ml when Ch/7-OOH-containing SUVs were used (Fig. 3). Thus, under the conditions described, 7-OOH exposure resulted in a substantial shortfall in testosterone output, suggesting significant cellular damage/dysfunction induced by 7-OOH.

### Cytotoxic Effects of 7-OOH on Primary Leydig Cells

**Fig. 4:**
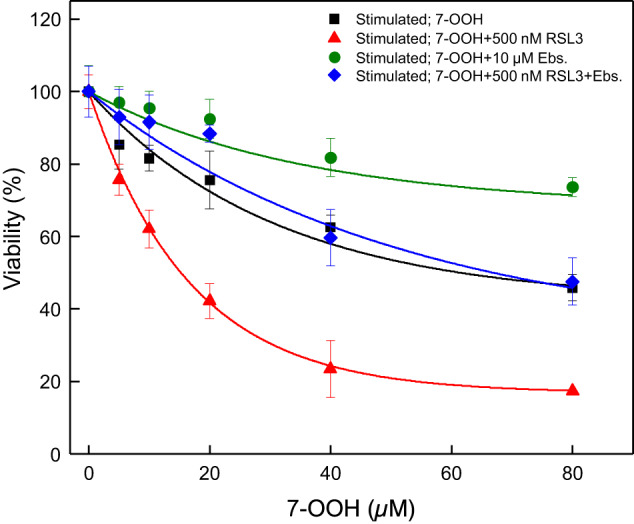
Cytotoxicity of liposomal 7-OOH to primary Leydig cells: cytoprotective effects of endogenous GPx4 or added Ebselen. Primary cells at 60–70% confluency in DMEM/F12 medium were stimulated with hCG (0.2 IU/ml) for 9 h, after which POPC/Ch (5:4 by mol) or POPC/Ch/7-OOH (5:4:1 by mol) SUVs were added, the former serving as a zero-peroxide control and latter giving the indicated concentrations of 7-OOH in bulk phase. Certain cell wells contained Ebselen alone (10 µM), RSL3 alone (500 nM), or RSL3 plus Ebselen from the outset. After 24 h of incubation in the presence of 1% serum, cell viability was determined by Neutral Rad assay. Plotted data represent cells in the absence of RSL3 or Ebselen (■), presence of RSL3 alone (), presence of Ebselen alone (), or presence of RSL3 plus Ebselen (); values are means ± SEM, *n* = 3

We turned next to isolated primary Leydig cells, which are more directly representative of the intact functional testis. As done with TM3 cells, we first assessed the cytotoxic range of 7OOH on primary cells before determining its effects on testosterone synthesis at sub-toxic or minimally toxic levels. As indicated by the data in Fig. 4, primary cells underwent a steady decrease in viability after incubation with 7-OOH in the presence of serum for 24 h, the drop-off at 20 µM peroxide being ~25%. When present during the 7-OOH challenge, RSL3 caused a striking additional drop-off, i.e. to ~40% viability at 20 µM peroxide. Although Ebselen provided no significant protection to TM3 cells (Fig. 1), it did protect primary Leydigs against 7-OOH, their viability decreasing only 5–10% with Ebselen present (Fig. 4). Importantly, Ebselen elicited a striking reversal of RSL3-enhanced viability loss, e.g. only ~20 % loss vs. ~60 % loss at 20 µM 7-OOH, suggesting that it was substantially counteracting the effects of GPX4 activity loss by RSL3.

### StarD1 and GPx4 Expression in Primary Cells: Effects of Stimulation ± Ch or Ch/ 7-OOH

**Fig. 5:**
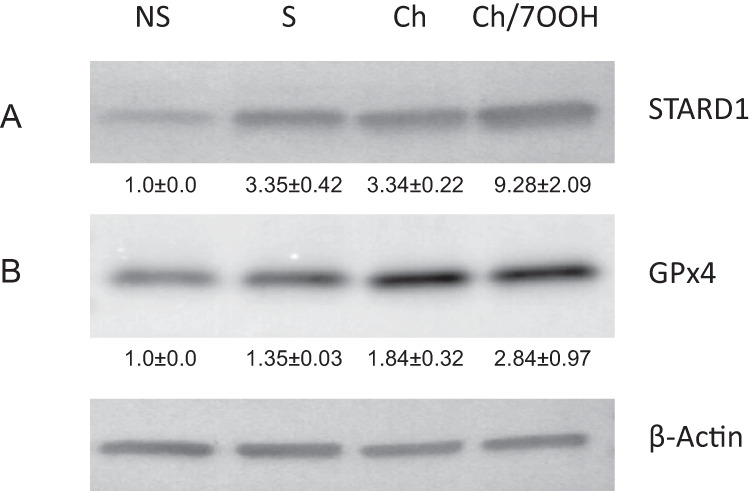
StarD1 and GPx4 expression in primary Leydig cells: effects of prior stimulation or stimulation followed by exposure to Ch or minimally cytotoxic Ch/7-OOH. Cells on 6-well plates were either not stimulated (NS) or stimulated (S) with LH (5 ng/ml) for 6 h, followed by 3 h incubation with POPC/Ch (5:4 by mol) SUVs (Ch) or POPC/Ch/7-OOH (5:4:1 by mol) SUVs (Ch/7-OOH; 10 µM overall peroxide in medium). Cells were then lysed and after measuring total lysate protein, StarD1 (A) and GPx4 (B) levels were determined by Western blotting

StarD1 and GPx4 expression after cell stimulation alone or stimulation followed by SUV-Ch or SUV-Ch/7-OOH exposure was assessed by Western blotting. Predetermined conditions resulting in little (if any) cell death during 7-OOH exposure were selected. As shown in Fig. 5A, LH stimulation resulted in a 3–4-fold upregulation of StarD1 relative to a non-stimulated control. Exposure to minimally lethal SUV-Ch/7-OOH (<5% cell death at 10 µM initial peroxide) resulted in a striking ~9-fold increase in StarD1 Fig. 5A. The indicated treatments with LH followed by SUV-Ch or SUV-Ch/7-OOH also resulted in GPx4 overexpression, its levels increasing 1.4-, 1.8-, or 2.8-fold over background, respectively (Fig. 5B). The upregulation of GPx4 after Ch of Ch/7-OOH exposure may be a pro-steroidogenic antioxidant response, i.e. for protection against any oxidative stress that impairs testosterone synthesis. The rather large StarD1 induction due to incubation with SUV-Ch alone is not surprising as a pro-steroidogenic reaction, but the larger induction due to UV-Ch/7-OOH (Fig. 5A) is difficult to explain, unless it somehow reflects the recently described role of StarD1 in maintenance of mitochondrial structure, function, and bioenergetics [46].

#### Testosterone Synthesis in Primary Leydig Cells: GPx4 Protection Against 7-OOH Effects

**Fig. 6:**
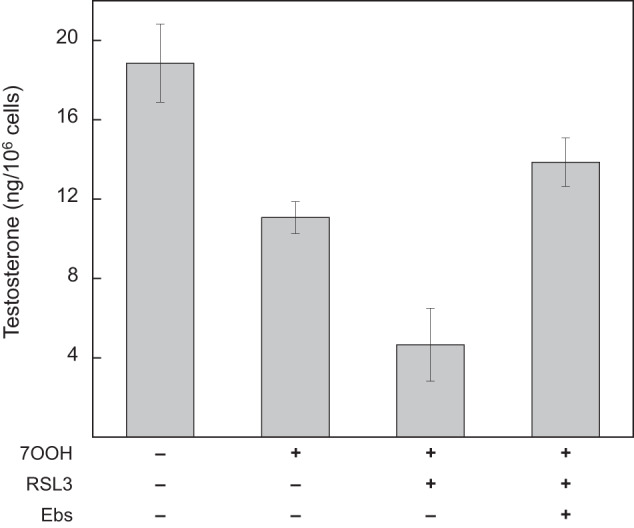
Testosterone production in stimulated primary Leydig cells: effects of 7-OOH exposure in the absence vs. presence of RSL3 or Ebselen. Cells in DMEM-F12 medium were stimulated with LH (5 ng/ml) for 6 h, then rinsed with fresh medium and exposed to POPC/Ch (5:4 by mol) or POPC/Ch/7-OOH (5:4:1 by mol) SUVs in the presence of RSL3 (0.5 µM) or Ebselen (10 µM). Initial 7-OOH concentration throughout was 20 µM in bulk phase. After a 3 h incubation, media samples were recovered for testosterone determination. Plotted values are means ± SEM (*n* = 3)

Based on our Fig. 5 findings, we predicted that exposing primary cells to 7-OOH would impair testosterone synthesis and that inhibiting or depleting GPx4 would exacerbate this. As shown in Fig. 6, testosterone generation by these cells dropped to ~60% of the control level after a 3 h incubation with SUV-Ch/7-OOH at 20 µM initial 7-OOH; cell survival was >95% under these conditions. When the GPx4 inhibitor RSL3 was present during incubation, testosterone output fell further to ~25% of the control, indicating that GPx4 had been strongly protecting these cells against 7-OOH-induced metabolic damage. However, when Ebselen was also present peroxide challenge, a large testosterone recovery to ~75% of control was observed (Fig. 6), indicating that that Ebselen had substantially “rescued” these cells from peroxide damage as it had done for TM3 cells (Fig. 2).

**Fig. 7:**
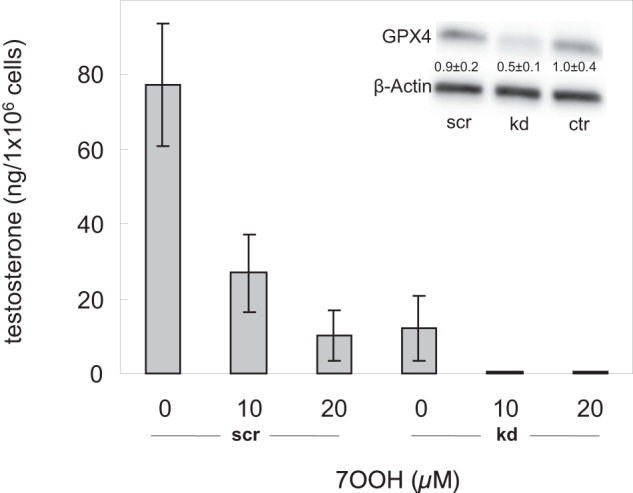
Effects of GPx4 knockdown on testosterone output of stimulated primary Leydig cells after a 7-OOH challenge. Cells were subjected to siRNA-based GPx4 knockdown (kd) as described in the *Methods* section; scrambled control cells (scr) were prepared alongside. *Inset*: Immunoblot showing average knockdown efficiency; numbers represent band intensities relative to β-actin and normalized to an untreated control. Bar graph depicts testosterone output of LH-stimulated GPx4-kd cells vs. scrambled controls after 2 h exposure to POPC/Ch/7-OOH (5:4:1 by mol) SUVs; starting overall 7-OOH concentrations are indicated. Final POPC and Ch concentration were constant for each reaction system. After 2 h of 7-OOH challenge, the medium was replaced with a fresh one, and testosterone accumulation over the following 12 h was measured. Plotted values are means ± SEM (n = 3)

In follow-up experiments, siRNA-based knockdown (kd) was used for depleting GPx4 prior to 7-OOH challenge. Extent of kd relative to scrambled control (scr) or wild type (wt) cells is shown by the immunoblot in the Fig. 7 inset. As indicated by the bar graph in Fig. 7, treating scr cells with increasing sub-toxic 7-OOH for 3 h resulted in a dose-dependent decrease in testosterone yield, which reached ~90% at 20 µM initial peroxide. GPx4-kd cells were much more sensitive, producing no measurable testosterone at 7-OOH > 10 µM. GPx4’s protective effects were also evident at zero 7-OOH, as shown by a > 80% drop in testosterone yield of kd cells compared with scr (Fig. 7). This illustrates the great importance of GPx4 to the metabolic well-being of these cells, even under wild type metabolic conditions.

#### GPx4-inhibitable lipid peroxidation in membranes of 7-OOH-treated primary Leydig cells

**Fig. 8:**
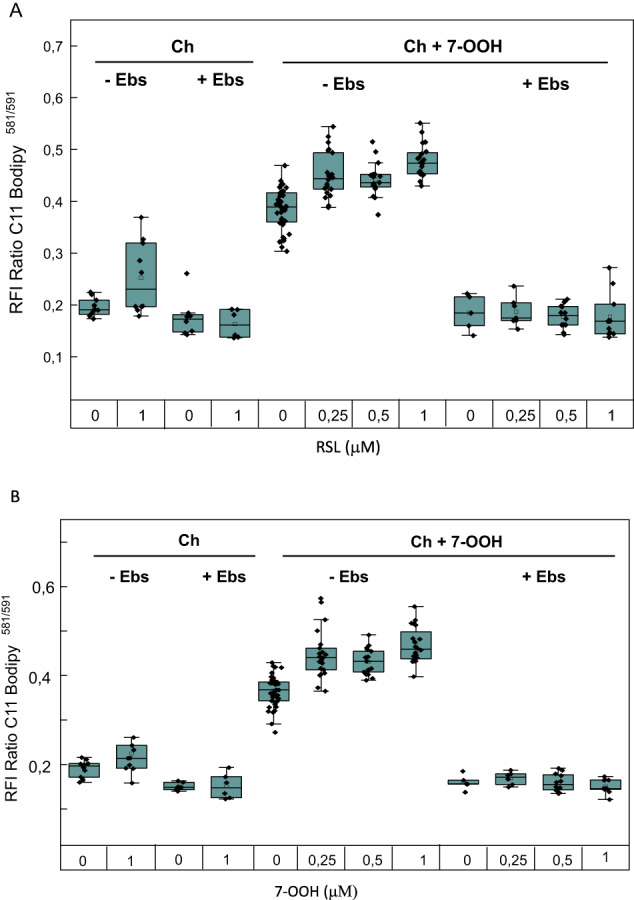
Free radical-mediated lipid peroxidation (LPO) in primary Leydig cells exposed to 7-OOH: effects of GPx4 inhibition. Cells on cover-glasses in 24-well plates were LH-stimulated for 6 h in the presence of RSL3 (0.5 µM) or RSL3 plus Ebselen, (10 µM) after which, MitoTracker Deep Red and C11-BODIPY-581/591 were added, giving final concentrations of 200 nM and 10 µM, respectively. After 45 min of labeling with these fluorophores, cells were washed and treated with POPC/Ch/7-OOH (5:4:1 by mol) SUVs at 20 µM 7-OOH in bulk phase. After 2 h of incubation, cells were examined by fluorescence microscopy, using LED sources for 625 nm or 470 nm excitation and bandpass filters for emission: 676/29 (MitoTracker); 605/15 (C11-BODIPY-581/591); and 525/30 for oxidized C11-BODIPY-581/591. (A) Overall cellular LPO; (B) Mitochondrial LPO. Plotted values are means ± SEM (*n* = 3)

Knowing that GPx4 can protect biological membranes against damaging chain LPO induced by one-electron turnover of primary LOOHs such as ChOOHs [22–24], we asked whether this was occurring in 7-OOH-challenged primary cells. To assess this, for Ch/7-OOH-challenged cells vs. Ch-only controls, we used the membrane-localizing fluorescent probe C11-BODIPY-581/591, which detects chain LPO [47]. As shown in Fig. 8A, which represents overall cellular LPO, control cells exhibited a significant increase in probe relative fluorescence intensity (RFI) when treated in the presence of RSL3. The RFI of Ch/7-OOH-treated cells was at least twice the control RFI and, again, RSL3 elevated it significantly. Each of these responses to RSL3 was abolished by Ebselen (Fig. 8A), suggesting that it could act as a GPx4-like antioxidant under the conditions used. We then focused specifically on mitochondria into which Ch, and presumably 7-OOH, are co-transported by StarD1 [30]. Overlap of C11-Bodipy-581/591 and MitoTracker Deep Red fluorescence was used to identify mitochondrion-centered LPO damage. As shown in Fig. 8B, the mitochondrial RFI of Ch/7-OOH-treated cells was substantially higher than that of controls. Once again, this signal was elevated by RSL3 in Ebselen-reversible fashion, further implicating GPx4 as an endogenous cytoprotective antioxidant in this system.

## Discussion

It is now well established that testis and other steroidogenic organs can become increasingly dysfunctional under physiological conditions associated with persistent oxidative stress with generation of reactive oxygen species [15–17]. Such dysfunction often occurs in conjunction with disorders such as chronic obesity with incipient type-2 diabetes, persistent inflammation, and atherogenesis. Steroidogenic dysfunction can also occur in elderly individuals and may be exacerbated by deficiencies in glutathione or selenium, or in vitamin E and other antioxidants [48]. Metabolically active Leydig cells in the testis acquire much of their Ch for testosterone synthesis from LDL in the circulation [5]. Under elevated oxidative pressure in vivo, significant amounts of LDL are converted to oxLDL. This oxLDL can be internalized by Leydig cells via their plasma membrane CD36 receptor [17]. Previous studies have shown that oxLDL contains measurable levels of redox active 7-OOH [25–27]. It is now clear that deficiencies in testosterone production and male fertility are linked to elevated levels of oxLDL in the blood circulation [15–17]. Thus, the level of oxLDL from oxidative stress correlates negatively with testosterone output.

**Fig. 9:**
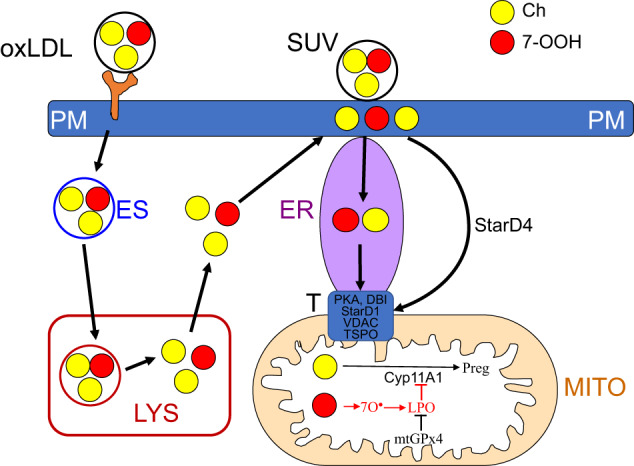
Scheme showing Leydig cell uptake of Ch- and 7-OOH-bearing oxLDL via the CD36 receptor. Endocytosed (ES) oxLDL is delivered to lysosomes (LYS), where Ch and 7-OOH are released and transferred sequentially to the plasma membrane (PM), then endoplasmic reticulum (ER), and finally to mitochondria (MITO). StarD1-containing transduceosome (T) delivers them to the inner membrane, where Ch undergoes Cyp11A1-catalzed conversion to pregnenolone (Preg) and 7-OOH suppresses this via GPx4-inhibitable LPO. Cytosolic StarD4 may also deliver Ch and 7-OOH to MITO, thereby bypassing ER. Delivery of liposome (SUV)-borne Ch and 7-OOH is also illustrated, whereby PM serves as the initial acceptor, then donor of these molecules

In the present study, we found that testosterone-generating TM3 and primary Leydig cells were highly sensitive to metabolic damage/dysfunction by 7-OOH at low concentrations, yet they remained viable. However, these cells did succumb to 7-OOH at relatively high concentrations. For delivering 7-OOH to Leydig cells, we used small unilamellar liposomes (SUVs) as relatively simple alternatives to oxLDL. We have extensive experience in using SUVs to expose various cell lines to ChOOHs [23, 24, 29, 30]. This study is the first to identify a specific ChOOH species generated by oxidative stress-induced free radical reactions as responsible for metabolic dysfunction in primary Leydig cells. To summarize, Ch+7-OOH-containing SUVs caused membrane LPO in primary cell mitochondria, which markedly reduced testosterone output. GPx4 knockdown or inhibition by RSL3 exacerbated these effects in Ebselen-reversable fashion, which is consistent with GPx4 being a major cytoprotective antioxidant in these cells. Based on these and previous findings [30], we suggest that Ch/7-OOH co-trafficking in the intact testis and GPx4 suppression of ensuing LPO can occur as illustrated in Fig. 9. Given that LDL, which interacts with the CD36 receptor, is a major source of Ch for testosterone synthesis by Leydig cells, oxLDL would supply 7-OOH as well as Ch. After entry in endocytic vesicles, the oxLDL is delivered to lysosomes, where Ch and 7-OOH are released and transferred to the plasma membrane [49]. They are subsequently delivered to the endoplasmic reticulum, and from there to the mitochondrial outer membrane, where StarD1 in the transduceosome complex [50] mediates final deposition at/near the inner membrane. Delivered Ch undergoes Cyp11A1-catalyzed conversion to pregnenolone, whereas delivered 7-OOH prevents this by triggering membrane damaging/disrupting LPO (Fig. 9). Mitochondrial GPx4 can inhibit this LPO by inactivating 7-OOH and/or downstream LOOHs. Cytosolic StarD4 may also deliver Ch and 7-OOH to mitochondria (Fig. 9), thereby bypassing endoplasmic reticulum. As shown in Fig. 9, and relevant to this study, delivery of liposome (SUV)-borne Ch and 7-OOH can also occur, whereby PM serves as the initial acceptor and then donor of these molecules.

The negative effects of 7-OOH trafficking to testis and other steroidogenic organs under pro-oxidant physiological conditions would be exacerbated by inadequacies in GPx4 level/activity or glutathione content. For GPx4 this might be ameliorated by dietary supplementation of selenium or possibly Ebselen administration. We look forward to testing these novel ideas in future studies, including those involving animal models.
